# Lipid and Glucose Serum Levels in Children with Congenital Heart Disease 

**Published:** 2014-01-12

**Authors:** Mehdi Ghaderian, Abdol Rahman Emami-Moghadam, Mohsen Ali Samir, Majid Amin Zadeh, Abdol Hamid Saadi

**Affiliations:** 1 *Emam Hosein Medical, Educational and Research Center, Esfahan University of Medical Sciences, Esfahan, Iran.*; 2 *Golestan Hospital, Jondi Shapoor University of Medical Sciences, Ahvaz, Iran.*; 3 *Aboozar Hospital, Jondi Shapoor University of Medical Sciences, Ahvaz, Iran.*

**Keywords:** Heart defects, congenital • Lipids • Glucose • Atherosclerosis

## Abstract

***Introduction:*** Coronary artery disease is one of the most common causes of morbidity and mortality in developed countries. Atherosclerosis begins in early childhood and progresses through life. With advances in pediatric cardiology, the prevalence of congenital heart disease in adults has increased in relation to children. A great deal of research has been conducted on serum glucose and lipid concentrations in patients with congenital heart disease, but comparison has yet to be made between congenital patients and the general population, especially in pediatric groups. The aim of this study was to compare the serum concentrations of glucose and lipids between pediatric congenital heart disease patients and a healthy age and sex-matched control group.

***Methods:*** We measured and compared the total cholesterol, low-density lipoprotein (LDL) cholesterol, high-density lipoprotein (HDL) cholesterol, triglyceride (TG), and plasma glucose concentrations of 100 pediatric congenital heart disease patients (cases) and 100 individuals matched for age and sex (controls) during a period of 7 months between November 2011 and June 2012.

***Results:*** Total cholesterol, triglyceride, HDL cholesterol, and LDL cholesterol concentrations were significantly higher in the patients than in the control group (p value < 0.05). Blood sugar levels in both groups had no significant difference (p value = 0.25). In the case group, the cholesterol level was higher in the males than in the females (p value = 0.30); moreover, the TG and HDL cholesterol levels were lower in the males than in the females and the LDL cholesterol and blood sugar levels had no statistically significant difference. In the control group, there was no difference between the males and females in terms of the cholesterol, HDL cholesterol, LDL cholesterol, TG, and blood sugar levels.

***Conclusion:*** The results of this study showed that our pediatric congenital heart disease patients had significantly higher levels of serum lipids than did their age and sex-matched controls. In light of these results, we recommend that the lipid profile be screened in children with congenital heart disease so as to reduce the risk of atherosclerosis.

## Introduction

Coronary artery disease is one of the most common causes of morbidity and mortality in developed countries. In the past, atherosclerosis was regarded as an adults’ problem and not a pediatric one. Atherosclerosis does not manifest itself clinically until the patient is in his middle ages; however, it begins in early childhood and progresses through life. Atherosclerosis and consequently coronary artery disease and stroke result from the accumulation and deposition of lipid and cholesterol in the arterial wall of the coronary and other arteries. High total cholesterol, increased low-density lipoprotein (LDL) cholesterol, reduced high-density lipoprotein (HDL) cholesterol, increased blood glucose (diabetes), cigarette smoking, obesity and overweight, metabolic syndrome, hypertension, and impaired physical activity and exercise can induce early lesions of coronary heart disease.^1^^-^^4^

With advances in pediatric cardiology, early physical examination and diagnosis, medical treatment, surgical methods, and postoperative care, the prevalence of congenital heart disease (CHD) in adults has risen in relation to children, and the number of adults living with CHD is expected to continue its upsurge.^5^ Now it is presumed that there are more adults with CHD living than there are children with CHD.^6^ Accordingly, control of lipid levels, serum glucose concentrations, and other risk factors has gained considerable significant.

For all the research conducted hitherto on serum glucose and lipid concentrations in CHD patients so far; comparison has yet to be made between CHD patients and the general population, not least in the pediatric population.^7^^-^^10^ To the best of our knowledge, the existing literature lacks a study that measures and compares serum levels of lipids between CHD children and normal children. Therefore, we conducted the present study to measure and compare the serum levels of lipids between children with CHD and an age and sex-matched control group.

## Methods

The study protocol was approved by the Ethics Committee of Jondi Shapoor University of Medical Sciences, Ahvaz, Iran, and the study was conducted during a 7-month period between November 2011 and June 2012. One hundred CHD patients (the case group) were compared with 100 age and sex-matched children (the control group). The case group was selected from the patients admitted to our Pediatric Congenital Heart Disease Unit or the Pediatric Cardiology Clinic of Golestan Hospital of Jondi Shapoor University of Medical Sciences, Ahvaz, Iran, for angiography or medical treatment. The control group was selected from a normal population who referred to our General Pediatric Clinic for simple diseases. 

The inclusion criterion for the case group was structural CHD diagnosed by echocardiography or angiography, and the inclusion criteria for both groups were comprised of age < 15 years and not having myeloproliferative disease, chronic liver, or kidney disease, which might affect lipid concentrations or glucose levels. None of the patients or children in the control group had received cholesterol-lowering drugs. All the subjects in both groups were born in the same region (relatively at sea level). 

The children and their parents/guardians received comprehensive explanations about the study (including procedural details and blood sampling), and written informed consent was obtained from the parents/guardians before the commencement of the study. 

For the measurement of total cholesterol, LDL cholesterol, HDL cholesterol, triglyceride (TG), and plasma glucose concentrations in milligrams per deciliter (mg/dl), blood samples were drawn from peripheral veins of the patients, who had been fasting for at least 4-6 hours, by a nurse experienced in the pediatric field. Levels of serum glucose and lipid were estimated via the spectrophotometric method in the Central Laboratory of Golestan Hospital by an experienced personnel member using one analyzer in order to have the same reference values and to avoid significant differences in the values. Total cholesterol, LDL cholesterol, HDL cholesterol, and TG were measured by the analyzer, and LDL cholesterol level was estimated via the Friedewald formula (LDL = total cholesterol − [HDL + TG/5]).

The subjects’ height and weight were measured using the standard method published in pediatric references. The Body Mass Index (BMI) was estimated as weight/height^2^. The subjects’ weight at birth was obtained from their hospital records. The CHD patients were classified by their major cardiac defects and by using basal O_2 _saturation, which was determined via pulse oximetry in rest (without using oxygen for classification to cyanotic or acyanotic CHD). Those with O_2 _≥ 92% were categorized as acyanotic CHD patients. All the patients underwent echocardiography or angiography (if necessary) for the classification of their CHD. The echocardiographic studies comprised two-dimensional, M-mode, and color Doppler using a GE Vivid 3 echocardiographic machine. The studies were performed in a quiet environment while the subjects were awake and non-sedated in a comfortable state. All the measurements were performed by one pediatric cardiologist.

The data are expressed as mean ± standard deviation (SD) and 95% confidence intervals. Differences in the variables between the case and control groups were compared using the *t*-test or the Mann-Whitney U test when appropriate. The Pearson χ^2^ test was used to study the association between the categorical variables. The Mann-Whitney U non-parametric test was utilized to compare two non-related variables, and the two-way analysis of variance (ANOVA) was employed to compare the lipid profiles and blood sugar levels between the males and females in the two groups. Different types of CHD, sex, age, and BMI were considered the independent variables in the present study. Statistical significance was defined as a p value < 0.05. For the statistical analyses, the statistical software SPSS version 18.0 for Windows (SPSS Inc., Chicago, IL) was used.

## Results

The lipid profiles and blood sugar levels of 100 consecutive children with CHD (admitted to the Pediatric Congenital Heart Disease Unit or the Pediatric Cardiology Clinic of Golestan Cardiovascular and Research Hospital at Jundi Shapour Ahvaz University of Medical Sciences, Iran, during a 7-month period between January 2012 and August 2012) were compared with those of 100 children from the normal population (referring to our General Pediatric Clinic for simple diseases). Table 1 shows the demographic, general, clinical, and analytical data of the cases and controls, and Table 2 illustrates the lipid and sugar levels of the case and control groups.

The case group included 22 patients with cyanotic CHD and 78 patients with acyanotic CHD. Ventricular septal defect (VSD), pulmonary stenosis (PS), patent ductus arteriosus (PDA), and atrial septal defect (ASD) were seen in 54, 14, 12, and 8 patients respectively. Aortic stenosis, coarctation of the aorta (COA), and complete atrioventricular septal defect (CAVSD) were seen in one patient separately. Tetralogy of Fallot (TOF) was seen in 6 patients, and other complex congenital heart diseases were seen in 3 patients. Eight patients had Down’s syndrome, which is associated with a congenital heart defect. In addition, ventricular septal defect was detected in 4 patients, atrioventricular septal defect in 2, patent ductus arteriosus in one, and atrial septal defect in one. Five patients had congenital heart defects in their siblings. One of these patients had cyanotic CHD and had a brother who had died at four months old (diagnosis of a single ventricle). Two patients were twins and had same-sized patent ductus arteriosus. One brother and one sister had aortic insufficiency. And finally, critical pulmonary stenosis was detected in 2 patients, one of whom died at in the postoperative period.

The serum cholesterol, HDL cholesterol, LDL cholesterol, and TG concentrations were significantly higher in the case group than in the controls (p value < 0.05). There was no statistically significant difference in the blood sugar levels between the two study groups (Table 2). In the case group, the cholesterol level was higher in the males than in the females (p value = 0.30); moreover, the TG (p value = 0.35) and HDL cholesterol (p value = 0.12) levels were lower in the males than in the females and the LDL cholesterol (p value = 0.62) and blood sugar (p value = 0.93) levels had no statistically significant difference. In the control group, there was no difference between the males and females in terms of the cholesterol, HDL cholesterol, LDL cholesterol, TG, and blood sugar levels. The two-way analysis of variance (ANOVA) confirmed our results and demonstrated that sex had no correlation with the lipid profiles and blood sugar levels in both groups and that CHD was correlated with the lipid profile and not with the blood sugar levels.

In the case group, those with Tetralogy of Fallot had the lowest TG and blood sugar levels. The difference, however, did not constitute statistical significance. With respect to the other lipid profile variables, there were no differences between the patients in the different subgroups. 

**Figure 1:**
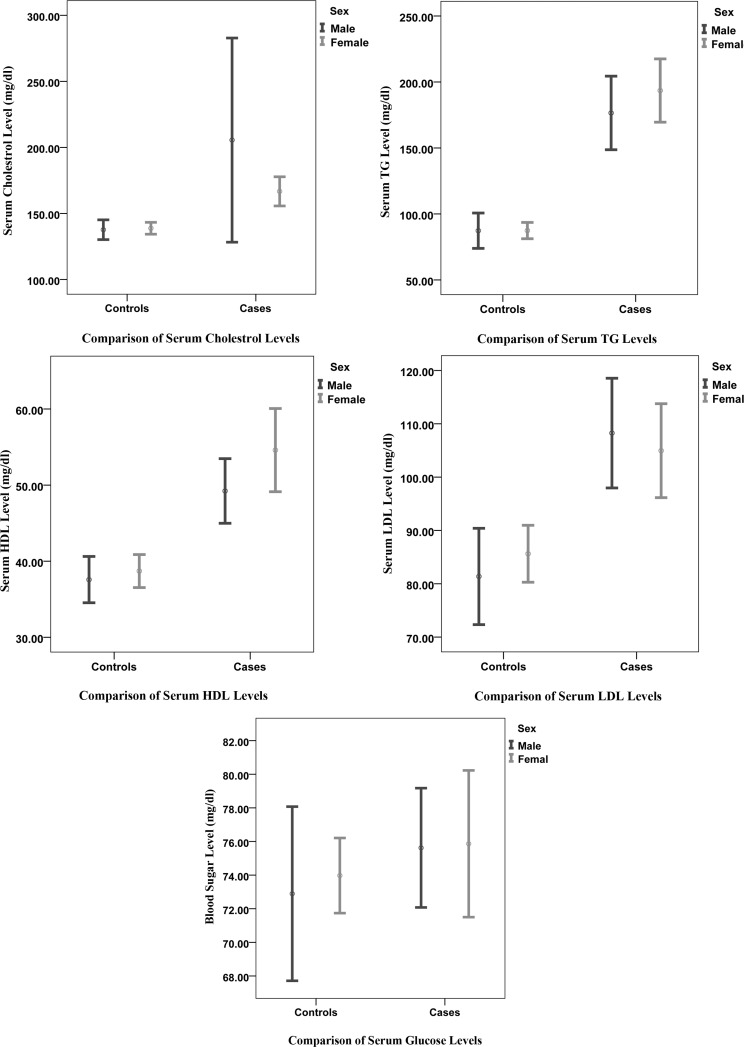
Comparison between males and females of case and control groups

TG, Triglyceride; HDL, High-density lipoprotein; LDL, Low-density lipoprotein

Comparison of the lipid profile between the cases and controls is depicted in Figure 1.

**Table 1:** Characteristics of the congenital heart disease group (case group) and the control group^*^

Variable	Case (n=100)	Control (n=100)	P value
Age (mo)	37.20±43.63	34.31±29.42	0.652
Sex (male)	48	45	-
Height (cm)	84.23±22.82	85.19±24.43	0.253
Birth weight (gr)	2878.18±40.34	3079.21±36.31	0.004
Weight (gr)	10613.12±60.42	11781.23±99.22	0.052
BMI (kg/m^2^)	14.64±3.35	14.74±3.29	0.822

BMI, Body mass index

* Date are presented as mean±SD

**Table 2:** Lipid and blood glucose serum levels in the males and females of the case and control groups

	Patients	Controls	P value
Cholesterol (mg/dl)			
Male	205.54±266.15	137.68±15.57	< 0.001
Female	166.72±39.67	138.77±20.26	< 0.001
Total	185.36±186.62	138.57±19.39	0.013
TG (mg/dl)			
Male	176.52±99.99	87.34±27.79	< 0.001
Female	193.50±80.50	87.39±27.94	< 0.001
Total	185.35±90.92	87.38±27.77	< 0.001
HDL (mg/dl)			
Male	49.22±14.69	37.57±6.30	0.021
Female	54.59±19.63	38.70±9.81	< 0.001
Total	52.02±17.52	38.49±9.22	< 0.001
LDL (mg/dl)			
Male	108.25±35.42	81.36±18.74	0.032
Female	104.95±31.64	85.62±24.14	< 0.001
Total	106.54±33.38	84.82±23.19	< 0.001
BS (mg/dl)			
Male	75.62±12.22	72.89±10.74	0.373
Female	75.86±15.67	73.79±10.10	0.392
Total	75.75±14.00	73.77±10.12	0.254

TG, Triglyceride; HDL, High-density lipoprotein; LDL, Low-density lipoprotein; BS, Blood sugar

## Discussion

CHD has an approximate incidence rate of 8 per 1000 live births and is, thus, deemed one of the most important causes of mortality in infancy and most common congenital defect at birth. In the past, many CHD patients could not reach adulthood, but recent advances in medical treatment, interventional approaches, surgical methods, and postoperative care in early life have resulted in a rise in the number of adults with such defects. It is now expected that more than 90% of patients born with CHD will survive to adolescence and adulthood. Currently, the number of adults with CHD is estimated to be equal to or more than that of children with CHD.^5^^, ^^6^

One of the most significant cardiovascular diseases is atherosclerosis, which begins in childhood and progresses slowly throughout life. An important and independent predictor of coronary heart disease is the lipid profile. This process relates to cholesterol concentration and accumulation and deposition of lipids and cholesterol in the arterial wall. One of the most important consequences of atherosclerosis is coronary heart disease due to the occlusion of the coronary arteries, leading to ischemic heart disease and myocardial infarction. Many factors such as overweight, hypertension, diabetes mellitus, smoking, and low physical activity are allied to an increase in lipid levels and consequently atherosclerosis.

Obesity and overweight in infancy and childhood is correlated with a rise in lipid profile levels in adolescence and adulthood and increased cholesterol concentrations and decreased HDL cholesterol.^11^^-^^13^ These changes are associated with an increase in the BMI. In our study, there was no significant difference in terms of the BMI between our two groups and it was not possible to make a comparison between the case and control groups regarding this index. 

Sex and age are two factors associated with a rise in total cholesterol and lipid profile levels in life, and adult studies have revealed that this rising trend begins in early life and continues until the fourth decade – whereupon it remains unchanged.^14^ In children between 5 to 10 years old, HDL is higher in boys than in girls. HDL levels increase in girls gradually and decrease in boys after this age through life. In our study, although in both groups the cholesterol, LDL cholesterol, HDL cholesterol, and TG levels were higher in the males than in the females (as is the case in the previous studies),^10^^, ^^15^^, ^^16^ the differences between the two groups did not reach statistical significance. 

In adolescent and young people, physical activity can influence the lipid profile and an increase in physical activity and exercise can reduce total cholesterol and TG levels. Vasconelos et al.^17^ and Barnard^18^ in separate studies reported that most adults could reduce serum lipid concentrations significantly by increasing their exercise. In our study, we believe that our case group patients – as compared with the controls – had limited physical activity, which was responsible for their worse lipid profile. 

We had no significant difference between the patients with Down’s syndrome and those without it. In contrast, some adult studies have reported that in Down’s syndrome patients, cholesterol and HDL cholesterol levels are decreased and TG levels are increased.^19^^, ^^20^ Patients with Down’s syndrome have a higher prevalence rate of type 1 diabetes mellitus and have higher blood sugar levels. None of our patients had diabetes mellitus; this could be in consequence of our small study size.

Research in adults with CHD has demonstrated that patients with cyanotic heart disease have lower total cholesterol and LDL cholesterol levels than their acyanotic counterparts.^7^^, ^^10^ It has been postulated that adults with CHD have hypoxic erythrocytosis and consequently lower cholesterol levels. In the fetal period, intrauterine hypoxemia does not influence fetal cholesterol; and before 6 months of age, fetal cholesterol is correlated with the mother’s cholesterol and after this age independent of maternal levels.^21^ In our study, we had no such results on account of the fact that the adults with cyanotic CHD had had chronic hypoxemia for a long time, whereas the children did not have such condition. Accordingly, the cholesterol and other lipid profiles were different between the adults and children. 

In other studies, patients with CHD had higher serum glucose concentrations,^10^ especially patients with left-to-right shunts such as those with atrial septal defects and ventricular septal defects due to the increased clearance of insulin by the lung tissue.^9^ In our study, there was no significant difference between the two groups as regards blood glucose. 

Hypercholesterolemia after correction by surgery and decrease of cyanosis tends to persist;^9^ this implies the suppression or induction of genes that decrease the lipid profile. In our study, we had no low cholesterol level. This theory requires further investigation. 

Our study on total cholesterol, LDL cholesterol, HDL cholesterol, and blood sugar levels is inconclusive because approximately all of our patients had only 4-6 hours of fasting and we could not have them fast for longer hours. According to our findings, however, there were statistically significant differences in regard to the lipid profile between our case and control groups.

Pediatric patients with CHD tend to have low physical activity and are likely to be fed with high-calorie formulas and foods in comparison with the normal population; these are factors that can exert a significant influence on the lipid profile. Another limitation of the present study is that our data could have been influenced if we had included patients at high risk of death from complex diseases.

To the best of our knowledge, the present study is the first study on the lipid profile in pediatric CHD patients. Further studies with larger sample volumes and longer follow-up durations should be carried out to investigate the influence of genetic and other factors on the lipid profile of children with CHD. Our findings suggest that the prevention of atherosclerosis in children with CHD is vitally important through better control of the lipid profile and feeding habits from early life. We propose that the lipid profile and atherosclerosis be screened from childhood.

## Conclusion

The cholesterol, LDL** c**holesterol, HDL cholesterol, and TG levels in our pediatric CHD patients were higher than those in their age and sex-matched controls; there was, however, no statistically significant difference between the study groups in terms of blood sugar. More studies with larger sample volumes are needed to confirm of our findings. 

## Notes

***This paper should be cited as:*** Ghaderian M, Emami-Moghadam AR, Samir MA, Amin Zadeh M, Saadi AH. Lipid and Glucose Serum Levels in Children with Congenital Heart Disease. J Teh Univ Heart Ctr 2013;9(1):20-26.
